# Embryonic Stem Cells Are Redirected to Non-Tumorigenic Epithelial Cell Fate by Interaction with the Mammary Microenvironment

**DOI:** 10.1371/journal.pone.0062019

**Published:** 2013-04-26

**Authors:** Corinne A. Boulanger, Robert D. Bruno, David L. Mack, Monica Gonzales, Nadia P. Castro, David S. Salomon, Gilbert H. Smith

**Affiliations:** 1 Mammary Stem Cell Biology Section, Cell and Cancer Biology Branch, National Cancer Institute, Bethesda, Maryland, United States of America; 2 Tumor Growth Factor Section, Mammary Biology and Tumorigenesis Laboratory, National Cancer Institute, Bethesda, Maryland, United States of America; The University of Texas M.D Anderson Cancer Center, United States of America

## Abstract

Experiments were conducted to redirect mouse Embryonic Stem (ES) cells from a tumorigenic phenotype to a normal mammary epithelial phenotype in vivo. Mixing LacZ-labeled ES cells with normal mouse mammary epithelial cells at ratios of 1∶5 and 1∶50 in phosphate buffered saline and immediately inoculating them into epithelium-divested mammary fat pads of immune-compromised mice accomplished this. Our results indicate that tumorigenesis occurs only when normal mammary ductal growth is not achieved in the inoculated fat pads. When normal mammary gland growth occurs, we find ES cells (LacZ+) progeny interspersed with normal mammary cell progeny in the mammary epithelial structures. We demonstrate that these progeny, marked by LacZ expression, differentiate into multiple epithelial subtypes including steroid receptor positive luminal cells and myoepithelial cells indicating that the ES cells are capable of epithelial multipotency in this context but do not form teratomas. In addition, in secondary transplants, ES cell progeny proliferate, contribute apparently normal mammary progeny, maintain their multipotency and do not produce teratomas.

## Introduction

In earlier publications, we demonstrated that dispersed mouse testicular, neural, bone-marrow-derived cells and mouse and human cancer cells were redirected to normal mammary epithelial cell fates when inoculated into epithelium-cleared mammary fat pads with normal mouse mammary epithelial cells (MEC's) [1]–[5]. Mouse embryonic stem cells (referred to as ES cells in these experiments), are derived from the inner cell mass of the blastocyst before germ layer formation occurs in the early embryo and are capable of forming all cell types of the developing and adult mouse [6]. Because of this unique potential, they can be used to identify developmentally relevant signals that pattern the embryo to form tissues and organs. Based on our understanding of somatic cell reprogramming [1]–[3] we sought to further investigate the potential dominant capacity of the mammary stem cell niche. The following experiments were designed to extend this observation by defining the inductive signals controlling this process by starting with the most undifferentiated stem cell, ES cells.

Using mouse ES cells *in vivo* is often troublesome due to their tumorigenic potential to form teratomas when injected into immune compromised hosts [7], [8]. Studies by G Barry Pierce showed that only the undifferentiated cells in these tumors give rise to teratomas, the differentiated cells do not [6], [7], [9]. Utilizing these undifferentiated cells allows evaluation of the mammary microenvironment's ability to reprogram embryonic cells that have not yet committed to a cell fate, and test the mammary gland's capacity to alter the teratoma-forming capability of mouse ES cells. Soriano [10], [11] developed mice designated ROSA Beta-geo 26 where expression of the Beta-geo reporter is constitutive during embryonic development. The embryonic stem cell cultures were derived from these mice. In these cultures LacZ expression marks all ES cells and makes them easily traceable in our experiments. Because LacZ expression is constitutive in these ES cells, our host animals do not need to be made pregnant prior to analysis as in previous experiments where WAP-Cre expression was the initiating activity [12]. The ES cells are grown on irradiated embryonic fibroblasts in the presence of leukemia inhibitory factor (LIF) to prevent differentiation. Here we demonstrate that the mammary microenvironment is sufficient to suppress ES cell induced tumorigenesis and to provide signals necessary to induce differentiation of ES cells to a mammary cell fate.

## Results

### MECs direct ES cells to adapt a mammary cell fate

**Figure 1:**
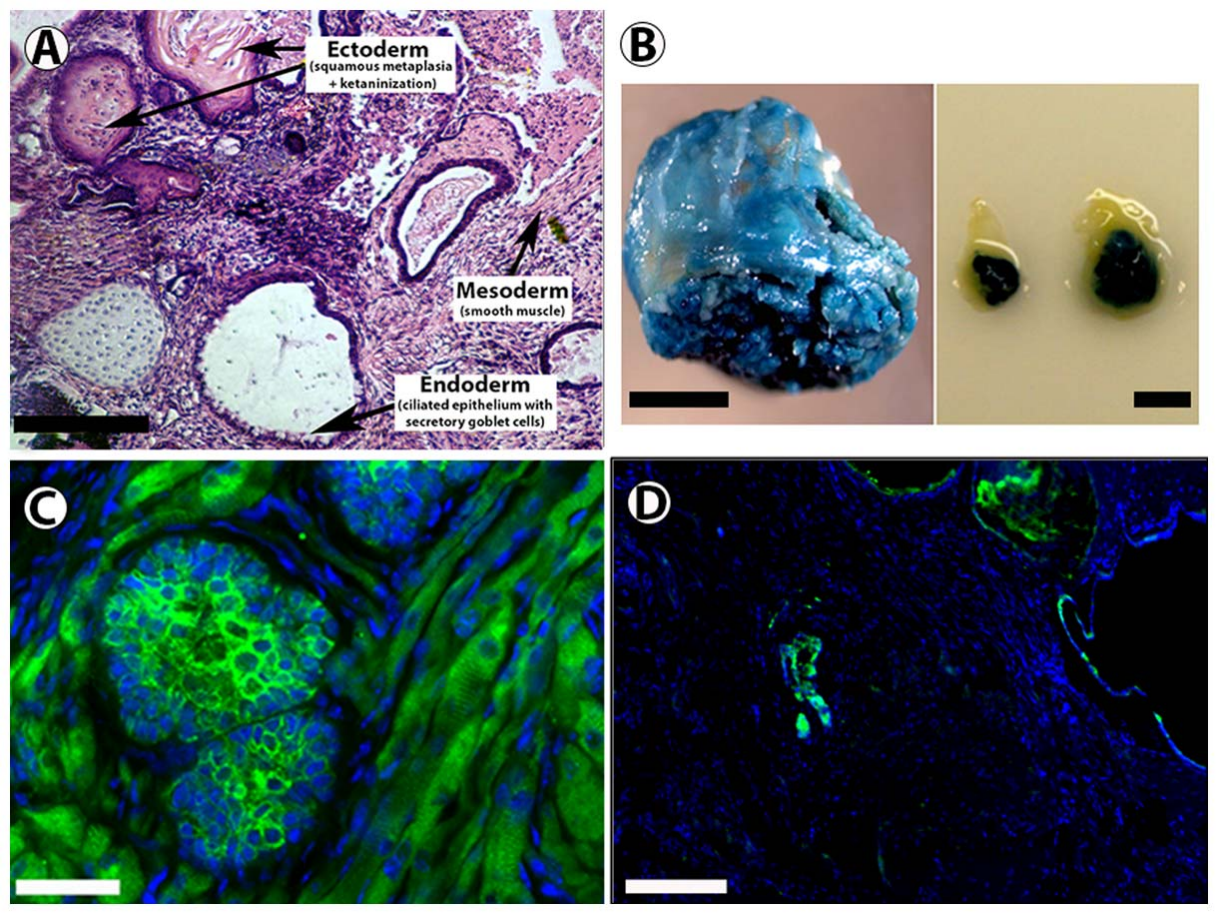
Embryonic stem (ES) cells give rise to teratomas that constitutively express Beta-gal when inoculated into cleared mammary fat pads. A) Cross section of ES derived tumors stained with hematoxylin demonstrates the presence of cell types from all three germ layers. B) ES cells give rise to tumors that stain positive for X-gal when inoculated into cleared mammary fat pads. C) Immunohistochemical staining with an anti-β-gal antibody (green) confirms expression of Beta-gal throughout the teratoma. D) Immunohistochemical staining with an anti-pan keratin antibody (green) reveals regions of the teratoma with epithelial like structures that express keratins. Scale Bars: A = 400 µM; B (right and left panels) = 5 mm; C = 100 µm; D = 400 µm.

When ES cells are transplanted into the cleared fat pad of nude mice, teratomas formed in all cases (Table 1, Fig. 1). As few as 1,000 (1 K) ES cells formed tumors in 4 out 4 transplants into cleared mammary fat pads. Histological analysis of teratomas shows evidence of the presence of all three germinal layers (Fig. 1A). The tumors constitutively expressed Beta-gal, confirmed by both X-gal staining (Fig. 1B) and immunofluorescence with an anti- Beta-gal antibody (Fig. 1C) and contained regions that expressed cytokeratins (Fig. 1D). Based on this data, and using information we obtained with other cancer reprogramming studies [4], [5], our mixing experiments were performed with 1 K and 10 K ES cells.

**Table 1:** Inoculation of ES cells.

# of ES Cells	# of MEC's	# LacZ+ mammary outgrowths/# inoculations	# Teratomas/# inoculations
50,000	0	0/5	5/5
10,000	50,000	5/13	3/13
10,000	0	0/4	4/4
1,000	50,000	8/13	1/13
1,000	0	0/4	4/4
2^nd^ generation	NA	6/10	0/10

ES cells are transplanted with or without MEC's. Teratomas were produced when ES cells were inoculated alone in all cases. When ES cells were mixed with MEC's normal growth is achieved for both 10 K and 1 K ES cells, and tumor incidence was reduced. These chimeric populations grew in second transplant generations with contributions by ES cells.

**Figure 2:**
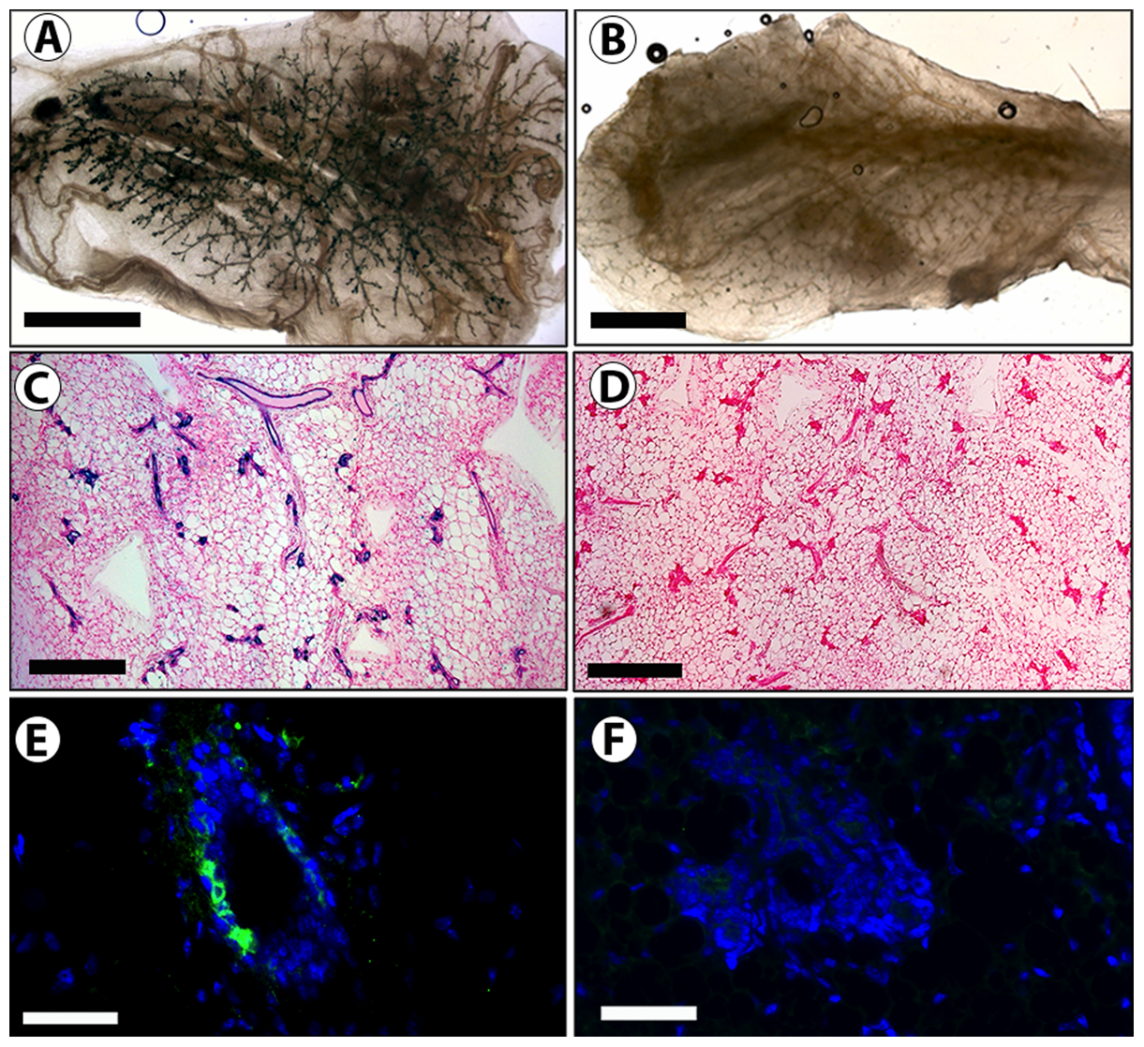
ES cells contribute to normal mammary outgrowths when inoculated with normal mammary epithelial cells. A and C) A whole mount (A) and cross section (C) of a representative chimeric outgrowth with positive X-gal stain (blue) resulting from the inoculation of a mixture of 1 K ES cells with 50 K mammary epithelial cells (MECs) into a cleared mammary fat pad (B and D) A whole mount (B) and cross section (D) of a representative control outgrowth with negative X-gal staining resulting from the inoculation 50 K MECs into a cleared mammary fat pad. E) Immunohistochemical staining with an anti-β-gal antibody confirms presence of Beta-gal expressing ES derived cells in mammary structures. F) Control mammary gland stained with the same anti-Beta-gal antibody. Scale Bars: A, B = 2 mm; C, D = 400 µm; E, F = 100 µm.

**Figure 3:**
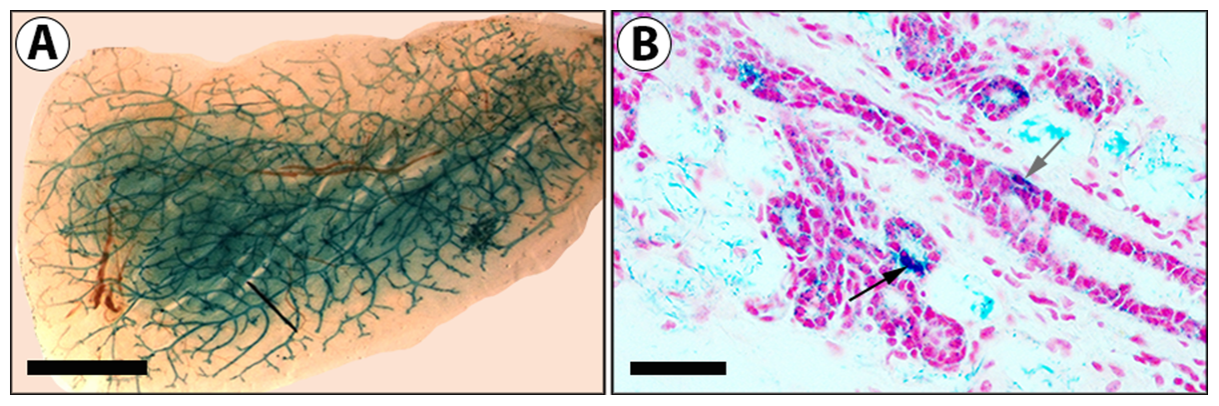
ES cells contribute to secondary mammary outgrowths. A) Mammary whole mount image of an X-gal positive stained outgrowth resulting from transplantation into a cleared mammary fat pad of a tissue fragment taken from a first generation ES and MEC chimeric outgrowth. B) Cross-section of a secondary outgrowth demonstrates presence of Beta-gal+ cells in mammary epithelial structures. Scale bars: A = 2 mm; B = 200 µm.

ES cells (1 K and 10 K) were mixed with 50 K mammary epithelial cells and inoculated into epithelial divested mammary fat-pads. Resulting morphologically normal mammary outgrowths contained cells derived from both populations as determined by the presence of both Beta-gal expressing (blue) and null cells (Table 1, Fig. 2). X-gal staining (Fig. 2 A–D), and immunofluorescent staining with an anti- Beta-gal antibody (Fig. 2 E–F) demonstrates that the ES cells are present and contribute to mammary structures. The ES derived cells were capable of self-renewal, evidenced by their ability to give rise to progeny present throughout the mammary epithelial tree of second-generation tissue transplants (Table 1, Fig. 3).

### Partial differentiation of ES cells does not significantly effect mammary reprogramming

To test whether differentiation of ES cells would enhance the formation of normal mammary chimeras between the pluripotent ES cells and mammary epithelial cells and blunt the appearance of tumors, ES cells were grown in the absence of LIF. ES cells that have been predisposed to differentiate into neuroectoderm and/or ectoderm due to LIF removal [13] may be more prone to differentiate into mammary epithelial stem or progenitor cells and less tumorigenic when placed into cleared mammary fat pads with mammary epithelial cells. To determine if partial differentiation of ES towards a neuroectodermal lineage would improve reprogramming efficiency, we removed LIF from the culture media of the ES cells for 7 days. Transcriptome analysis of ES cells that had been grown in the absence of LIF demonstrated up-regulation of genes or signaling pathways known to be required for embryonic mammary placode development, such as the WNT (Wnt4, Wnt10b), FGF (Fgf9, FgfR1, FgfR2), Hedgehog (Gli3), Nrg3, keratins (Table 2). In addition, genes such as Otx2 and Pax6, believed to be involved in neuroectoderm and/or ectoderm differentiation [14] [15], [16] were also up regulated. Conversely, ES cells grown in the absence of LIF down-regulated expression of genes associated with pluripotency and differentiation inhibition such as Nanog, Cripto/Tdgf1, Sox2, Lefty1/2, and Nodal (Table 2) [17]–[19]. Together, these results suggested that the removal of LIF had successfully driven partial differentiation of the ES cells away from a totipotent state and towards an ectoderm/neuroectoderm lineage.

**Table 2:** Genes Differentially Regulated in ES cells grown in the absence of LIF.

	Fold Change LOG relative to MEFs	
Down-regulated Genes	ES wild type	ES without LIF
Bmp4	4.27	−1.74
Eed	4.18	2.58
Fgf4	2.20	−0.42
Fgf7	8.93	3.17
Gdf3	4.84	0.93
Lefty1	3.45	−0.32
Lefty2	5.05	−1.74
Nanog	2.70	−2.00
Nodal	3.83	−0.23
Tbx2	4.63	1.72
Tbx3	4.02	−1.74
Sox2	4.31	1.17
Cripto/Tdgf1	3.66	−4.06

Up-regulated Genes	ES Wild type	ES without LIF
Fgf9	2.58	6.04
FgfR1	5.08	6.44
FgfR2	3.47	6.81
Gli3	4.64	6.67
Krt8	1.83	6.82
Krt14	0.85	3.58
Krt18	1.77	6.55
Nrg3	5.34	7.02
Otx2	3.22	5.09
Pax6	4.93	6.32
Prom1	2.19	6.13
Wnt4	4.21	7.89
Wnt10b	2.77	6.17

However, as shown in Table 3, removal of LIF did not have an effect on the tumorigenic potential of ES cells implanted into cleared mammary fat pads. Teratomas arose from both LIF treated and untreated ES cells, and the resulting teratomas exhibited similar transcriptomes (Table S1). Furthermore, LIF removal failed to elicit a statistically significant difference (p = 0.1780 according to Fisher's 2×2 Exact Test) in ES cell response to the mammary microenvironment (as measured by presence of ES derived cells in morphologically normal mammary outgrowths). Therefore, the mammary microenvironment (fatty stroma and epithelium) is sufficient to induce ES cell differentiation. Prior in vitro differentiation of ES cells towards a neuroectoderm/ectodermal cell fate had little to no effect on their interaction with MEC to form chimeric mammary outgrowths or on teratoma formation.

**Table 3:** ES cells without LIF inoculations.

# of ES cells	# of MEC's	# LacZ+ mammary outgrowths/# inoculations	# Teratomas/# inoculations
10,000	0	0/2	2/2
10,000	50,000	5/7	2/7
1,000	0	0/2	2/2
1,000	50,000	4/5	1/5

Data from ES cells grown in the absence of LIF. This allows ES cells to begin to differentiate. When this occurs cells contribute to chimeric outgrowths and retain their teratoma forming capacity.

### Identification of ES cell progeny in chimeric outgrowths

**Figure 4:**
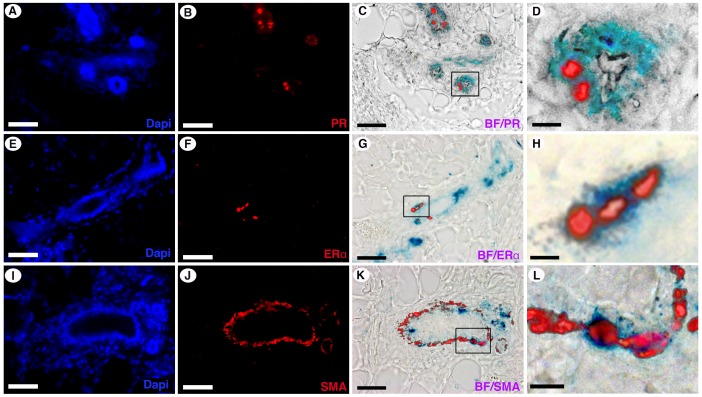
ES cells differentiate into ERα positive, PR positive, and SMA positive cell types. Immunofluorescent staining for PR (A–D), ER-alpha (E–H), and SMA (I–L) on chimeric outgrowths from inoculations of 1,000 ES cells with 50,000 MECs. ES cell expression of PR (C and D), ER-alpha (G and H), and SMA (K and L) is demonstrated by overlay of a bright field (BF) image of the X-gal stain (marking ES cells) with an image of the immune-fluorescent staining of the antigen. Panels D, H, L are enhanced images of the boxed region shown in panels C, G, and H, respectively. Scale Bars: A–C, E–G, I–K = 100 µm; D, H, L = 20 µm.

To determine if ES progeny in chimeric outgrowths had undergone complete differentiation to a mammary epithelial cell fate, sections from the outgrowths were subjected to immunohistochemical staining for estrogen receptor alpha (ER-alpha), progesterone receptor (PR) and smooth muscle actin (SMA) (Fig. 4 A–L). Cells were located which were doubly positive for X-gal and PR (Fig. 4A–D), for X-gal and ER-alpha (Fig. 4E–H), and for X-gal and SMA (Fig. 4,I–L). These results demonstrate ES cell progeny differentiated into three mammary epithelial cell subtypes (ER-alpha/PR-positive and ER-alpha/PR-negative luminal cell and myoepithelial cells) when interacting with normal mouse mammary epithelial cells during gland regeneration in a cleared mammary fat pad.

## Discussion

The reforming mammary gland in epithelium-free mammary fat pads has been shown to redirect adult stem/progenitor cells from other organs and tissues to mammary epithelial cell fates [1]–[3]. In previous studies with tumor cells in our laboratory, both mouse and human cancer cells were reprogrammed and produced non-tumorigenic progeny in regenerating mammary glands when mixed with normal mammary epithelial cells [4], [5]. To test whether this effect was manifest in mixtures of tumorigenic totipotent ES cells and mouse mammary epithelial cells, we utilized mouse embryonic stem cells that constitutively express E. coli beta-galactosidase. This allowed us to readily identify ES cell progeny by cell fate mapping in either tumors or in chimeric mammary outgrowths. In cleared inguinal fat pads of nude mice, transplanted ES cells alone formed teratomas in all cases, with as few as 1 K cells. When ES cells were mixed with wild type mouse mammary epithelial cells before inoculation into the mammary fat pads of host mice, they uniformly either formed teratomas or were reprogrammed and contributed non-tumorigenic mammary epithelial cell epithelial progeny of various phenotypes to mammary epithelial outgrowths. This was true when either 10 K or 1 K ES cells were mixed with 50 K normal MEC. Larger number (50 K) ES cells contributions invariably produced teratomas in spite of the presence of 50 K normal MEC. The embryonic mesenchyme that subsequently becomes the mammary fat pad in the adult mouse has the capacity to act as an inductive mesenchyme for the full development of a variety of embryonic epithelial organs in tissue recombinant experiments [20], [21]. This capacity is lost at 17 days post-coitus in utero and subsequently this organ only supports the complete development of the mammary epithelial gland and the hair follicle [21]. In our experiments reported here, we introduced mouse embryonic cells into the adult mammary fat pad at various cellular quantities from cultures, which had been maintained in LIF, to ensure the presence of fully pluripotent embryonic cells, and in the absence of LIF to allow enhanced differentiation of the embryonic cells in vitro. In all cases the ES cells produced teratomas when inoculated by themselves, regardless of the presence or absence of LIF in culture. When normal adult MEC were mixed with ES cells, mammary outgrowths were produced relatively often where ES cells had contributed progeny during the regeneration of the mammary epithelium. This was unaffected by the presence or absence of LIF in the medium the week before implantation. We speculate that interaction of ES cells with mammary epithelial cells leads to differentiation of the ES cells towards a mammary epithelial cell fate. Our evidence suggests that ES cells differentiate into both luminal and basal (myoepithelial) mammary epithelial cells. Recent publications indicate that both luminal and basal mammary epithelial cells can contribute progeny to mammary epithelial outgrowths upon transplantation [12], [22]. Thus interaction of ES cells with either luminal or basal mammary epithelium may result in their differentiation along these distinct mammary epithelial cell fates. Our results indicate that interaction of ES cells with each other in the context of the mammary fat pad often leads to tumorigenesis.

Fragments of the original chimeric mammary outgrowths were implanted to form second-generation transplants. The ES cell progeny continued to behave as non-tumorigenic mammary epithelial cells and contributed to all portions of developing glands. These results were interpreted to mean that the ES cells were able to stably occupy the reformed niches in the regenerated mammary gland, and function within these niches to give rise to fully differentiated progeny during secondary growth, and be self-renewed and persist in second transplant generations in the absence of teratoma formation.

When ES cells were allowed to differentiate in culture (in the absence of LIF), there was no effect on the tumorigenic potential of ES cells, or the transcriptome of the resulting teratomas. There was also no statistically significant effect on the ability of the mammary microenvironment to induce ES cell differentiation to a mammary epithelial cell fate. It is possible further differentiation of ES cells (to the point where they no longer form teratomas) may affect reprogramming efficiency, but it is clear from these results that interaction with the regenerating mammary microenvironment is, in most cases, sufficient to direct ES cells to adopt a mammary epithelial cell fate and not teratomas.

We did not observe an inhibition or suppression of ES cell tumorigenesis when ES cells were mixed with MEC at a 1∶1 (50 K∶50 K) ratio. We hypothesize that interaction between ES cells leads to a rapid production of teratoma, which overgrows any evidence for mammary outgrowth. As the ratio between ES cells and MEC is decreased to 1∶5 or 1∶50 the chances that ES cells interact with MEC is increased and their interaction with other ES cells is decreased. We hypothesize that this interaction is important to tumor suppression.

## Materials and Methods

All mice were housed in Association for Assessment and Accreditation of Laboratory Animal Care–accredited facilities in accordance with the NIH Guide for the Care and Use of Laboratory Animals. The National Cancer Institute (NCI) Animal Care and Use Committee approved all experimental procedures.

### Cell and tissue transplantation

Mammary fat pad clearing and transplantation was performed on female mice between 3 and 4 weeks of age as previously described [23]–[26]. Briefly, mice were anesthetized and endogenous epithelium was removed from the #4 and #9 inguinal fat pads by surgically removing the proximal portion (from the nipple to the lymph nodes). Mammary tissue fragments, (1–2 mm^2^), were directly inserted into a small cavity in the fat pad created with watchmaker forceps. Dispersed cells were suspended in 10 µl of DMEM without serum per injection and inoculated into cleared mammary fat pads with a Hamilton syringe equipped with a fine 30-gauge needle. Tissue and cell transplants were then allowed to grow for 12 weeks before harvesting of the glands. Differences in gland formation and tumorigenesis efficiencies were evaluated using a two-tailed 2×2 Fisher's Exact Test.

### Mouse Embryonic Fibroblast (MEFs) and Rosa 26 ES cell Preparation

Mouse embryonic Rosa 26 ES cells, [27] (A gift from P. Soriano) were maintained in the undifferentiated state by culture on irradiated mouse embryonic fibroblast (MEF) (Cat. # 56×2. ATCC, Manassas, VA) feeder layers in high-glucose DMEM supplemented with 15% fetal bovine serum, 0.1 mmol/L 2-mercaptoethanol, 1 mmol/L sodium pyruvate, 1× nonessential amino acids, 2 mmol/L glutamine, 100 U/ml penicillin/streptomycin, and 1000 U/ml murine leukemia inhibitor factor (LIF). Undifferentiated ES cells growing on mitotically inactivated embryonic fibroblasts (irradiated with total of 60Gy of gamma irradiation) were detached with ES medium containing 1.5 mg/ml collagenase IV. The collected ES cells were then incubated with 0.25% trypsin solution and seeded at 2×10^5^ cells in 60-mm plates that had been pre-coated with 0.1% gelatin.

### Mammary Epithelial Cell Preparation

Mammary epithelial cells are isolated according to standard primary cell culture protocol as follows: Glands were minced into 1–2 mm fragments and incubated overnight at 37°C in complete medium (DMEM, 10% fetal bovine serum, 1.0 µg/ml porcine insulin, 1.0 ng/ml EGF, 1.0% penicillin/streptomycin) containing 1 mg/ml Type 1A collagenase (Sigma Aldrich, St Louis MO). The suspension was then triturated through a 10 ml pipette 3 times and the cells were pelleted by centrifugation for 10 minutes at 300× g. The pellet was then washed with twice with an equal volume of complete medium without collagenase and sheared through a 19-gauge needle one time. The resulting organoids were pelleted as before and suspended in 15 ml complete media and cultured in T-75 flask for 3–4 days under normal cell culture conditions (37°C/5% CO2). Differential trypsinization was performed to remove fibroblasts prior to collection of epithelial cells.

### X-Gal Staining and whole mounts of Mammary Glands

Glands were spread on glass slides, fixed in paraformaldehyde (4.0%) for 1–2 h, and permeabilized in 0.01% Nonidet P-40, 0.01% sodium deoxycholate and 2 mM MgCl2 in phosphate buffer saline overnight at 4°C. Glands were then stained with X-gal (5-bromo-4-chloro-indolyl-β-D-galactopyranoside) staining solution (5 mM potassium ferricyanide crystalline (K_3_Fe(CN)_6_); 5 mM potassium ferricyanide trihydrate (K_4_Fe(CN)_6_.3H2O) 1 mg/ml X-gal) for 24–30 hours at 37°C in an incubator. Glands were then washed in PBS and post-fixed with Carnoy's fixative overnight at 4°C. For whole mounts, glands were then dehydrated in stepwise treatment with ethanol starting with 2×70% for 30 min., and then 2×100% for 60 min. When fully dehydrated, the whole mounts were cleared in xylenes for 20 minutes, mounted on glass slides and cover slipped with Permount (Fisher cat. # SP15-100) for stereo-microscopic analysis at 5–20X.

### Immunohistochemistry

X-gal-stained whole mounts (above) were embedded in paraffin, sectioned at 6.0 μm, and counterstained with nuclear fast red (Sigma-Aldrich, St Louis, MO, USA). Immunofluorescence was performed on deparaffinized sections. Sections were blocked with 10% normal goat serum, or 5% BSA+0.1% Gelatin (for chicken anti-betagalactosidase) in TBS+0.1% Triton X-100. Primary antibodies used were rabbit anti-ER-alpha MC-20 (1∶50; Santa Cruz Biotechnology, Santa Cruz, CA, USA), rabbit anti-PR (1∶75; Dako, Carpinteria, CA, USA), mouse-anti-SMA 1A4 (1∶100; Zymed/Invitrogen, Carlsbad, CA, USA), chicken anti-beta-galactosidase (1∶100, Abcam, Cambridge MA, USA). Secondary antibodies used were Alexafluor 568 goat anti-rabbit IgG (Invitrogen), Alexafluor 568 goat anti-mouse IgG (Invitrogen), and FITC rabbit anti Chicken IgY (Abcam). Antigen retrieval was performed by boiling sections for 20 min in pH 9.0 Tris-EDTA+0.05% Tween-20. Sections were mounted with Prolong Gold Antifade plus Dapi (Invitrogen).

### RNA isolation and quality

Total RNA from ES cells was isolated using QIAshredder^TM^ (Cat. #79656; Qiagen-Germantown MD); RNeasy Mini kit (Cat. #74106; Qiagen-Germantown MD) and DNase treated using the RNase-Free DNase Set (Cat. #79254; Qiagen-Germantown MD) in accordance with the manufacturer's instructions.

Total RNA RNA from teratomas were isolated using TRIzol Reagent (Cat. #15596-018; Life Technologies). RNA quality was assessed by RNA integrity using the Agilent RNA 6000 Pico Chip on the Agilent 2100 Bioanalyzer (RNA 6000 Nano LabChip, Agilent Technologies, Santa Clara, CA, USA).

### Real time RT-PCR analysis of a customized gene array

The single strand cDNA from 1μg of total RNA was synthesized using RT2 first strand kit (Cat. #330401- Qiagen-Germantown MD). Real-Time PCR was performed according to the User Manual of RT2 Profiler PCR array system (SABioscience/Qiagen -Germantown MD) using RT2 SYBR Green ROX qPCR Mastermix (Cat. #330520- Qiagen-Germantown MD). Thermal cycling and fluorescence detection were performed using an Mx3005P Detection System (Stratagene). A customized array was designed containing genes involved in embryonic stem cell state and mammary placode development. Data were analyzed using RT2 Profiler PCR Array Data Analysis version 3.5 (Table S1).

## Supporting Information

Table S1 **Genes Differentially Regulated in ES/MEC transplants.** ES cells grown in presence or absence of LIF.
(DOCX)
