# Supplementary material for: Embryonic Stem Cells Are Redirected to Non-Tumorigenic Epithelial Cell Fate by Interaction with the Mammary Microenvironment
Source: PLoS One. 2013 Apr 26;8(4):e62019. doi: 10.1371/journal.pone.0062019 (PMC3637449; doi:10.1371/journal.pone.0062019)
Supplement: Table S1 — Genes Differentially Regulated in ES/MEC transplants. ES cells grown in presence or absence of LIF. (DOCX) [file pone.0062019.s001.docx]

**Supplementary Table 1.** Genes Differentially Regulated in ES/MEC transplants. ES cells grown in presence or absence of LIF.

_______________________________________________________________________ Fold Change LOG relative to ES wild type cells

Teratomas derived from:

**Down-regulated Genes** ES with LIF/MEC ES without LIF/MEC

| Bmp4 | -3.5 | -3.21 |
| --- | --- | --- |
| Eed | -3.15 | -3.1 |
| Fgf4 | -12.3 | -10.5 |
| Fgf7 | -2.82 | -4.6 |
| Fgf17 | -4.96 | -6.38 |
| Foxd3 | -4.34 | -7.27 |
| Foxh1 | -8.97 | -9.38 |
| Gdf3 | -3.35 | -6.86 |
| Lefty1 | -6.01 | -5.67 |
| Lefty2 | -8.29 | -10.97 |
| Nanog | -6.57 | -6.98 |
| Nodal | -6.89 | -6.30 |
| Otx2 | -3.48 | -4.01 |
| Pou5f1 | -18.93 | -16.03 |
| Sox2 | -4.00 | -6.00 |
| Tbx3 | -5.54 | -7.64 |
| Cripto/Tdgf1 | -10.7 | -13.29 |
| Utf1 | -10.70 | -13.29 |

**Up-regulated Genes**

| Acta2 | 3.60 | 2.41 |
| --- | --- | --- |
| Afp | 6.54 | 3.22 |
| Bmp3 | 6.35 | 3.64 |
| Csf1r | 8.74 | 7.47 |
| Dkk4 | 3.57 | 3.03 |
| Esr1 | 5.99 | 4.31 |
| Fgf9 | 4.54 | 5.19 |
| FgfR1 | 2.81 | 1.28 |
| FgfR2 | 4.99 | 3.97 |
| Gata6 | 3.95 | 1.02 |
| Gfap | 12.49 | 9.94 |
| Krt5 | 11.57 | 11.69 |
| Krt6a | 11.87 | 9.26 |
| Krt8 | 4.48 | 3.51 |
| Krt14 | 11.4 | 9.44 |
| Krt17 | 3.21 | 2.54 |
| Krt18 | 5.26 | 2.79 |
| Krt19 | 5.97 | 4.75 |
| Msln | 6.78 | 3.96 |
| Nrg3 | 2.20 | 1.60 |
| Msx1 | 3.60 | 2.72 |
| Pax6 | 2.87 | 5.89 |
| Pgr | 4.22 | 1.97 |
| Prom1 | 5.78 | 4.58 |
| Pthlh | 4.90 | 3.60 |
| Pthr1 | 3.21 | 3.23 |
| Runx1 | 4.14 | 2.35 |
| Shh | 6.32 | 4.69 |
| Tbx2 | 4.72 | 3.98 |
| Trp63 | 8.45 | 6.43 |
| Wnt4 | 4.98 | 1.84 |
